# The Modern Treatment of Wounds

**Published:** 1882-01

**Authors:** 


### THE MODERN TREATMENT OF WOUNDS.

The November number of the *Proceedings of the Medical Society of Kings* is taken up by a discussion upon the treatment of wounds, in the form of well-written essays by several authors. Dr. J. D. Rushmore opens the discussion by a paper on *the process of healing*.

He goes very extensively into the general conditions which influence the healing of wounds, because they sometimes fail to receive the attention they deserve from surgeons. The author thinks that Esmarch's bandage is a questionable advantage, inasmuch as he has seen three cases of paralysis resulting after its use. He also objects to it on account of the prolonged oozing which follows its use. It is not stated whether the paralysis in all the cases referred to was permanent. He prefers the ligature over the other methods of controlling hemorrhage. He uses adhesive plaster instead of sutures when possible, and prefers horsehair to other materials for sutures. The rubber drainage-tube with the side perforations has seemed to act better than other varieties of drainage-tubes. After the third or fourth day the wound does better without drainage-tubes.

*The Treatment of Open Wounds* is considered by Prof. J. S. Wight. He says that after arrest of hemorrhage, removal of foreign bodies, coaptation of cut surfaces and covering contused surfaces with carbolized lint, the surgeon may obtain more or less speedy formation of scar tissue.

This constitutes open treatment of wounds as differing from Listerism, and is based on the principles of antiseptic surgery.

After stating that incised wounds are included under this treatment, the author makes the point that simple antiseptic treatment, based on disinfecting the surroundings of the patient, produces as good results as Listerism.

The outbreak of *Sepsis* in Long Island College Hospital, in 1873, is referred to. After wards, building, &c., had been thoroughly disinfected the trouble continued. But when the hands of the attendants and the instruments were disinfected the outbreak ceased.

After relating a case which shows how important is the general condition of the patient, how age, nutrition and alcoholic poisoning influence the occurrence of sepsis, he urges forcibly the removal of effete matters from the system. "For as the yeast cell cannot live in alcohol, the nutrition cell cannot live on excretion."

The writer tabulates the indications favorable to the formation of scar tissue as follows:

"1st. The complete arrest of hemorrhage.

"2nd. Most perfect rest, both local and general.

"3d. The most complete removal of waste products, bloody serum, purulent fluids and the excreta.

"4th. The coaptation of fresh or granulating surfaces.

"5th. The proper protection of the part injured, preventing irritation and keeping the tissues at a proper temperature.

"6th. The most perfect cleanliness of the patient and his environment."

Prof. A. J. C. Skene deals in a comprehensive manner with the *Treatment of Wounds in Gynæcology.* He confines himself to wounds of the uterus and perineum.

The author regrets that on account of position we can not apply, with perfect success, the principles of antiseptic surgery to these wounds.

Among the conditions which retard the healing of these, lactation and involution are mentioned as having special bearing.

It is claimed that warm water acts as well as cold in arresting hemorrhage, and that it does not have the same bad influence on the healing of the wound. As an antiseptic dressing to wounds of the uterus a tampon of borated cotton is recommended.

Dr. George R. Fowler, contributes an essay on *The Listerian Treatment of Wounds.*

This is largely an historical article in which Listerism is presented in its most favorable light.

The wonderfully more favorable results after the introduction of this method especially in the German hospitals, is referred to at length.

After reading this no one can doubt that Lister has produced a great revolution in the treatment of wounds.

